# Effects of in-center daily hemodialysis upon mineral metabolism and bone disease in end-stage renal disease patients

**DOI:** 10.1590/S1516-31802001000300004

**Published:** 2001-05-02

**Authors:** Jocemir Ronaldo Lugon, Mauro Barros André, Maria Eugênia Leite Duarte, Simone Martins Rembold, Elisa de Albuquerque Sampaio da Cruz

**Keywords:** Daily hemodialysis, Renal osteodystrophy, Bone disease, Hemodiálise diária, Osteodistrofia renal, Doença óssea

## Abstract

**CONTEXT:**

Alternative hemodialysis schedules have been proposed to improve the quality of the dialysis. Nonetheless, their influence upon mineral and bone disorders is unknown.

**OBJECTIVE:**

To report the impact of a daily hemodialysis schedule upon the lesions of renal osteodystrophy.

**TYPE OF STUDY:**

Prospective non-controlled study.

**SETTING:**

Public University Hospital.

**PARTICIPANTS:**

Five patients treated by daily hemodialysis for at least 24 months.

**INTERVENTION:**

Daily dialysis sessions were accomplished with non-proportional dialysis machines without an ultrafiltration control device, with blood flow of 300 ml/min, bicarbonate dialysate ([Ca]=3.5 mEq/L) at 500 ml/min, and low-flux membrane dialyzers. Sessions were started at 6:00 p.m. (except Sundays) and lasted 2 hours.

**MAIN MEASUREMENTS:**

Serum levels of Ca and P from the last 6 months on conventional hemodialysis for the same patients were used for comparison with each semester of daily hemodialysis. Bone biopsies and PTH levels were obtained at the end of the conventional hemodialysis period and then again after 2 years of daily hemodialysis.

**RESULTS:**

Mean serum calcium was significantly higher during the second and third semesters of daily dialysis [10.0 mg% (SD 0.6), and 10.0 mg% (SD 0.8), respectively] compared to standard dialysis [9.4 mg% (SD 0.8)], p < 0.05. Mean values for phosphorus were significantly lower during every semester of daily hemodialysis [6.3 mg% (SD 1.8), 5.8 mg% (SD 1.7), 6.0 mg% (SD 1.7), and 6.0 mg% (SD 1.8)] compared to standard dialysis [7.2 mg% (SD 2.7)], P < 0.05. Variations in mean Ca x P product followed the same pattern as for phosphorus [59.5 (SD 16.0), 57.1 (SD 16.3), 59.8 (SD 17.7), and 58.31 (SD 20.9) vs. 68.6 (SD 27.3), P < 0.05]. After 2 years on daily hemodialysis, 2 patients who had aplastic lesion were found to have mild bone disorder. In addition, one patient with mixed bone lesion and moderate bone aluminum accumulation had osteitis fibrosa with no aluminum. Intact PTH values at the beginning of study and after 2 years on daily hemodialysis did not differ [134 pg/ml (SD 66) vs. 109 pg/ml (SD 26), P = 0.60, respectively].

**CONCLUSIONS:**

Patients treated using daily hemodialysis had better control of serum phosphorus and perhaps a lower risk of metastatic calcifications. Daily hemodialysis also seemed to be beneficial to low turnover bone disease and bone aluminum deposition.

## RESUMO

**CONTEXTO:**

Diversos esquemas alternativos de hemodiálise foram propostos para melhorar a qualidade da diálise. Apesar disso, sua influência sobre o metabolismo mineral e a doença óssea permanece desconhecida.

**OBJETIVO:**

Relatar o impacto de um esquema diário de hemodiálise sobre as lesões da osteodistrofia renal.

**TIPO DE ESTUDO:**

Estudo prospectivo não controlado.

**LOCAL:**

Hospital Universitário Público.

**PARTICIPANTES:**

Cinco pacientes tratados por hemodiálise diária por pelo menos 24 meses.

**INTERVENÇÃO:**

As sessões foram realizadas com máquinas-tanque sem dispositivo controlador de ultrafiltração, fluxo de sangue de 300ml/min, banho de bicarbonato ([Ca] = 3.5 mEq/L) a 500 ml/min, e dialisadores com membrana de baixo fluxo. As sessões eram iniciadas às 18 horas (exceto domingos) e duravam duas horas.

**VARIÁVEIS ESTUDADAS:**

Os níveis séricos de Ca e P, dos últimos seis meses em hemodiálise convencional dos mesmos pacientes, foram usados para comparação com cada semestre em hemodiálise diária. As biópsias ósseas e os níveis de PTH foram obtidos no fim do período convencional em hemodiálise e após dois anos em hemodiálise diária.

**RESULTADOS:**

O cálcio sérico médio era significativamente mais elevado durante os segundo e terceiro semestres da diálise diária (10.0 DP 0.6 mg%, e 10.0 DP 0.8 mg%, respectivamente) em comparação à diálise convencional (9.4 DP 0.8 mg%), P < 0.05. Os valores médios de fósforo foram significativamente mais baixos durante cada semestre em hemodiálise diária (6.3 DP 1.8 mg%, 5.8 DP 1.7 mg%, 6.0 DP 1.7 mg%, e 6.0 DP 1.8 mg%) comparados aos da diálise convencional (7.2 DP 2.7 mg%), P < 0.05. As médias do produto CaxP acompanharam o padrão observado para o fósforo (59.5 DP 16.0, 57.1 DP 16.3, 59.8 DP 17.7, e 58.31 DP 20.9 vs. 68.6 DP 27.3, P < 0.05). Após dois anos em hemodiálise diária, dois pacientes que tinham lesão aplástica mudaram para lesão óssea inicial do hiperparatireoidismo secundário; Além disso, um paciente com lesão mista e deposição óssea de alumínio evoluiu para osteíte fibrosa leve sem alumínio. Os valores do PTH intacto no começo do estudo e após dois anos na hemodiálise diária não diferiram (134 DP 66 vs. 109 DP 26 pg/ml, P = 0.60, respectivamente).

**CONCLUSÕES:**

Os pacientes em hemodiálise diária tiveram melhor controle do fósforo sérico e, talvez, menor risco de calcificações metastáticas. Além disso, também mostrouse benéfica à doença óssea de baixo turnover e atenuou a deposição óssea de alumínio.

## INTRODUCTION

Mineral metabolism abnormalities and bone disease are frequently found in end-stage renal disease patients undergoing standard hemodialysis (3 sessions of four hours each per week).^1–3^ On the other hand, alternative hemodialysis schedules like short high-flow hemodialysis,^4–6^ high-efficiency hemodialysis,^7,8^ slow long-duration hemodialysis^9,10^ and daily hemodialysis,^11,12^ have recently been proposed in an attempt to improve the quality of the dialysis offered. In this regard, particularly impressive data have been reported using daily hemodialysis.^11,13^ However, the impact of the different schedules of hemodialysis upon the mineral metabolism disorders and bone disease remains obscure.

The present report derives from a prospective study, partially reported elsewhere,^14^ and designed to assess the effect of a long period of in-center short-duration daily hemodialysis upon complications affecting end-stage renal patients.

## METHODS

This was a prospective non-controlled study, which ran for a period of two years. Five male patients were recruited from a standard hemodialysis program developed in a public University Hospital. The decision to enter the study was a free option following explanations about the protocol. There was no restriction on the type of primary renal disease. Strategy regarding management of hyperphosphatemia and dietary intake during standard and daily dialysis was kept the same. Daily dialysis sessions were accomplished with the same equipment and strategy used for conventional hemodialysis in the center. They consisted of non-proportional mixture machines without an ultrafiltration control device, with blood flow of 300 ml/min, bicarbonate buffered dialysate ([Ca]=3.5 mEq/L) at 500 ml/min, and low-flux membrane dialyzers. A de-ionizer was used to provide water treatment. The daily sessions were accomplished from Monday to Saturday and began at 6:00 pm lasting 2 hours. Routine biochemical tests (urea, creatinine, albumin, calcium, and phosphorus) were performed monthly. Biochemical data from each of the same patients relating to the last 6 months on standard hemodialysis (3 sessions of 4 hours each per week) were used for comparison with data taken from each semester of daily hemodialysis. Bone biopsies were obtained at the end of the standard hemodialysis period and 2 years after the start of daily hemodialysis. Serum levels of intact PTH were obtained at the beginning of the study and then again at the end of the follow-up period of 2 years.

Kt/V was calculated in a middle-of-week-session by the formula -logN (R-0.008t-0.75*UF/W) in which R is the ratio of post and pre dialysis serum urea, t is the duration of the dialysis session, UF is the ultrafiltration during the session and W is the post-dialysis body weight. It was expressed as weekly Kt/V to allow comparison between standard and daily hemodialysis and adjusted for the missing days. Post-dialysis urea was collected from the arterial line four minutes after reduction of dialysate flow to zero. Levels of urea, creatinine, albumin, calcium and phosphorus were determined by an autoanalyzer (Cobas Mira, Roche, Switzerland) and intact PTH by immunoradiometric assay.

### Bone Biopsy

The study patients underwent anterior iliac crest bone biopsy under local anesthesia. Prior to the bone biopsy, patients were given two three-day courses of tetracycline separated by an interval of 10 days. The biopsy was performed 4 days after the last dose of tetracycline. All biopsy specimens were fixed in 70% ethanol and dehydrated by sequential changes in ascending concentrations of ethanol and xylene and then embedded in methyl methacrylate. For histological analysis, undecalcified sections (5m) of cortical and trabecular bone were stained by the Goldner method^15^ and by aurine-tricarboxylic acid for detection of aluminum.^16^ Bone remodeling and turnover was investigated on 10m unstained sections under fluorescent light.

The bone biopsies were evaluated by standard histomorphometric methods as previously described^17,18^ using the terminology established by the Nomenclature Committee of the American Society of Bone and Mineral Research.^19^

### Statistics methods

Data are shown as mean and standard deviation. Repeated variance analysis measurements complemented by the Duncan test were used to compare dependent ordinal data. Category variables were compared by the Chi-square test. Values of P less than 0.05 were considered significant.

## RESULTS

Patients were all male (four white and one black), aged 41 years (SD 12), and had been on standard dialysis for 50 months (SD 21) (Table 1). Primary renal diseases were: malignant nephrosclerosis (n=3), chronic glomerulonephritis (n=1), and diabetic nephropathy (n=1).

**Table 1:** Characteristics of patients

Number of patients		5
Sex, M/F.		5/0
Age, years.		41 (SD 12)
Race, B/W.		1/4
Time on dialysis, months.		50 (SD 21)
Primary renal disease		
	Malignant nephrosclerosis	3
	Chronic GN	1
	Diabetic nephropathy	1

They were regularly receiving vitamin supplements and anti-hypertensive drugs as needed. One patient had received human recombinant erythropoietin and another one had received calcitriol while on standard hemodialysis. Hyperphosphatemia was managed with calcium carbonate or calcium acetate (short courses of aluminum hydroxide were used whenever necessary). Depending upon availability in the center, dialyzers belonged to the CF-series and CA-series (Baxter Healthcare Co.), Clirans-series (Terumo Co.) or the low flow members of the F-series family (Fresenius USA Inc.). The percentage utilization of dialyzers by studied patients during the standard dialysis period was 37% for CF-25 (non-modified cellulose, 1.6 m^2^, Kuf = 6.5 ml/mmHg/hour), 27% for F-6 (polysulfone, 1.2 m^2^, Kuf = 5.5 ml/mmHg/hour), 20% for C-121 (cuprophane, 1.2 m^2^, Kuf = 6.1 ml/mmHg/hour), 8% for CF-23 (non-modified cellulose, 1.25 m^2^, Kuf = 5.2 ml/mmHg/hour), 6% for F-7 (polysulfone, 1.6 m^2^, Kuf = 7.2 ml/mmHg/hour), and 2% for CA-170 (cellulose acetate, 1.7m^2^, Kuf = 8.3 ml/mmHg/hour). Corresponding values for the daily dialysis period were: 45% for CF-25, 35% for F-7, 8% for F-5 (polysulfone, 1.0 m^2^, Kuf = 4.2 ml/mmHg/hour), 7% for C-121, and 5% for CF-23. Dialyzer reuse for standard and daily periods were 17 times (SD 4), and 16 times (SD 4), respectively.

Mean urea levels during standard dialysis were 164 ± 34 mg%. Values were significantly lower (P < 0.05) in each semester of daily dialysis, reaching 117 mg% (SD 32) in the last set of measurements (Table 2). A similar pattern was found for mean creatinine levels [9.3 mg% (SD 1.2) and 8.3 mg% (SD 1.3) for standard dialysis and the fourth semester of daily dialysis, respectively]. In contrast, mean albumin levels were significantly (P < 0.05) and uniformly higher during daily dialysis. The initial value of 4.1 g% (SD 0.4) in standard dialysis increased to 4.3 g% (SD 0.3) at the end of the study period. Values for Weekly Kt/V tended to be higher for each semester of daily in comparison to standard dialysis but significance was only found for the second semester [4.13 (SD 1.57) vs. 3.12 (SD 0.50), P < 0.05].

**Table 2:** Serum level of urea, creatinine, and albumin, and Kt/V during standard and daily hemodialysis

	Standard	Daily			
		Semester 1	Semester 2	Semester 3	Semester 4
Urea, mg%.	164^a^(34)	126* (30)	120* (31)	119* (30)	117* (32)
Creatinine, mg%.	9.3 (1.2)	8.5*(0.9)	8.3*(1.0)	8.2* (1.0)	8.4* (1.3)
Albumin, g%.	4.1 (0.4)	4.4*(0.3)	4.3*(0.3)	4.3*(0.3)	4.3* (0.3)
Kt/V b, weekly.	3.12 (0.78)	3.86 (1.79)	4.1 3*(1.57)	3.49 (1.43)	3.78 (1.95)

a *Mean, standard deviation in parenthesis;*

b *Adjusted for missing days (2% and 9% for standard and daily dialysis, respectively);*

* *P < 0.05 vs. Standard dialysis*

Serum levels of calcium, phosphorus, and Ca x P product during standard and daily hemodialysis are shown in Table 3. Values for mean calcium were significantly higher (P < 0.05) during the second and third semesters of daily dialysis [10.0 mg% (SD 0.6), and 10.0 mg% (SD 0.8), respectively] in comparison with values from standard dialysis [9.4 mg% (SD 0.8)] and with values from the first and fourth semesters of daily dialysis [9.5 mg% (SD 0.8), and 9.4 mg% (SD 0.7), respectively]. On the other hand, mean values for phosphorus were significantly lower (P < 0.05) during every semester of daily hemodialysis [7.2 mg% (SD 2.7) vs. 6.3 mg% (SD 1.8), 5.8 mg% (SD 1.7), 6.0 mg% (SD 1.7), and 6.0 mg% (SD 1.8) for standard dialysis and first, second, third and fourth semesters of daily dialysis, respectively]. Variations in mean Ca x P product followed the same pattern as those from phosphorus with values consistently lower during daily dialysis [68.6 (SD 27.3) vs. 59.5 (SD 16), 57.1 (SD 16.3), 59.8 (SD 17.7), and 58.31 (SD 20.9) for standard, and first, second, third, and fourth semester of daily dialysis, respectively].

**Table 3:** Serum levels of calcium, phosphorus and Ca x P product during standard and daily hemodialysis

	Standard	Daily				
		Semester 1	Semester 2	Semester 3	Semester 4
Calcium, mg%.	9.4 (0.8)	9.5 (0.8)	10.0*(0.6)	10.0*(0.8)	9.4 (0.7)
Phosphorus, mg%.	7.2 (2.7)	6.3# (1.8)	5.8# (1.7)	6.0# (1.7)	6.0# (1.8)
Ca x P, both in mg%.	68.6 (27.3)	59.5# (16.0)	57.1# (16.3)	59.8# (17.7)	58.31# (20.9)

*Standard deviation in parenthesis;*

* *P < 0.05 vs. Standard dialysis, and semesters 1 and 4 of daily dialysis;*

# *P < 0.05 vs. Standard dialysis.*

When values for calcium and phosphorus were analyzed for the presence of episodes of hypercalcemia and hyperphosphatemia, and for the risk of metastatic calcifications, the frequency of Ca≥10.5 mg/dl was significantly higher (P = 0.05) during the second and third semesters of daily dialysis (30% for both sets of measurements) in comparison to the first and fourth semesters of daily dialysis (7% for both sets of measurements) (Table 4). The frequency of P ≥ 7.0 mg/dl tended to be lower during daily dialysis but statistical significance was not found (p = 0.45). A similar pattern was observed for the frequency of the Ca x P product ≥ 70 (p = 0.52).

**Table 4:** Episodes of hypercalcemia and hyperphosphatemia, and risk of metastatic calcifications (Ca x P) during standard and daily hemodialysis

	Standard	Daily				
		Semester 1	Semester 2	Semester 3	Semester 4
Ca ≥ 10.5 mg%	3	2	9*	9*	2
P ≥ 7.0 mg%	14	8	8	10	11
Ca x P ≥ 70, both in mg%.	13	7	8	9	9

* *P < 0.05 vs. Standard and semesters 1 and 4 of daily dialysis.*

Bone biopsies were obtained from the five patients at the start of the study and then again after two years of daily dialysis in four of the five patients (Table 5). Before daily hemodialysis, three patients had low turnover (aplastic bone disorder), and one had mixed bone lesion with stainable aluminum deposits covering 55% of the trabecular surface. Following two years of daily hemodialysis, two of the three patients with aplastic bone disorder were found to have mild bone lesion. The bone of the diabetic patient remained adynamic. The patient with mixed bone lesion and moderate aluminum accumulation was found to have mild osteitis fibrosa with no significant aluminum deposition in bone.

**Table 5:** Bone biopsy findings and serum PTH levels before daily hemodialysis and after 2 years of follow-up

Patients	Hemodialysis	
	Standard	Daily
	Bone biopsy/PTH (pg/ml)	Bone biopsy/PTH (pg/ml)
1	Aplastic bone disorder/185	Mild bone disorder^/^124
2	Aplastic bone disorder/66	Mild bone disorder/138
3	Aplastic bone disorder/90	Aplastic bone disorder/98
4	Mixed bone lesion^a^/195	Mild osteitis fibrosa/79
5	Mild osteitis fibrosa/200	-/103

a *positive staining for aluminum.*

Intact PTH values from the beginning of daily hemodialysis and the ones collected after 2 years on daily hemodialysis did not differ [134 pg/ml (SD 66) vs. 109 pg/ml (SD 26), P = 0.60, respectively).

## DISCUSSION

Renal osteodystrophy is a multifactorial disorder of bone remodeling that frequently affects hemodialysis patients.^3^ In the last few years different hemodialysis schedules have been proposed as alternatives to improve the dialysis treatment.^20^ In this setting, short duration daily hemodialysis has been reported to improve quality of life, increase hematocrit and albumin, and allow easier control of blood pressure.^11,13^ The effect of this hemodialysis regime upon the bone disease itself, however, has not been addressed before.

Consistent with previous reports,^11^ many of our findings indicate that, on the whole, the efficacy of the dialysis procedure improved when the in-center short-duration daily hemodialysis schedule was adopted. Both urea and creatinine pre-dialysis levels decreased substantially. Although statistically significant only during the second semester, increases in the weekly Kt/V were seen in each semester. The nutrition of our patients as assessed by their serum albumin levels also experienced some improvement in spite of no change in dietary recommendations. All this occurred within the same contextual framework of 12 hours of dialysis per week, characteristic of standard dialysis.

Before the study, the effect of the daily hemodialysis regime upon the serum levels of calcium and phosphorus represented a special concern. The daily exposure to a dialysate with a calcium concentration of 3.5 mEq/L could potentially contribute to an increased frequency of hypercalcemic episodes, thus favoring metastatic calcifications. In fact, a minor but significant increase was observed in the serum levels of calcium during the second and third semesters in daily dialysis, when the frequency of hypercalcemic episodes reached 30%. By the fourth semester, however, mean serum calcium values were close to the values recorded at the beginning of the study and the frequency of hypercalcemic episodes was reduced to 7%. The fluctuations in the serum calcium during the study could be accounted for by the patient's voluntary dietary adjustment or by modifications in their hormonal profile. However, no significant difference was found between the levels of intact PTH analyzed at baseline and those recorded at the end of the study period, a time when, unfortunately, the corresponding serum levels of calcium were also very close.

During the study period, mean serum levels of phosphorus from each semester were lower than those from the baseline. However, different from the experience with nocturnal dialysis,^21^ phosphate binders could not be discontinued. Indeed, undesirable levels of phosphorus were frequently found even during daily dialysis. Nonetheless, serum levels of phosphorus were low enough to keep the mean Ca x P product within a range safer than during standard dialysis, perhaps at a lower risk of metastatic calcifications. The frequency of a high Ca x P product (^3^ 70) during daily dialysis was lower than that observed during standard dialysis, but statistical significance was not found. By design, the strategy regarding management of hyperphosphatemia by dietary adjustments and phosphate binders during standard and daily dialysis was kept the same. However, the possibility that more strict adherence to the clinical recommendations may have played some role in the lower phosphorus levels observed in patients on daily dialysis can not be entirely discarded.

Four patients underwent a second bone biopsy after two years of daily dialysis, which is thought to be the gold standard for diagnosis of renal osteodystrophy. In only one patient whose primary renal disease was diabetic nephropathy did the bone histology remained unchanged, with aplastic bone disorder being found in both bone samples. In the remaining patients, there was a noticeable change in the histological findings. In two of the patients with previous findings of low turnover bone disorder, the predominant histological findings in the second biopsy were of mild hyper-parathyroidism. In the fourth patient, there had been resolution of the osteomalacic component of the mixed lesion of renal osteodys-trophy and almost complete elimination of the aluminum deposits from the bone (the residual aluminum was not found to affect either bone formation or mineralization).

It should be stressed that these changes took place in a context of no treatment with calcitriol, as the only patient using this compound refused the second bone biopsy. These findings suggest that the low-turnover component of the renal osteodystrophy is in some way related to the uremic environment. Skeletal resistance to trophic factors affecting the bone had previously been imputed to the uremic state and implicated in the genesis of renal osteodystrophy.^22,23^ This potential patho-physiological aspect of mineral abnormalities in end-stage renal patients could certainly be ameliorated by intensification of dialysis. This hypothesis is consistent with the report of a higher prevalence of low-turnover bone disease in patients treated by continuous outpatient peritoneal dialysis,^2^ a method in which the dose of delivered dialysis is more restricted. Whether the higher extraction of medium molecules by daily dialysis that was previously reported^24^ has any role in this setting is still unknown. The almost complete elimination of the bone aluminum in the patient with mixed bone disease, in the absence of desferrioxamine treatment, is another relevant finding that could also be related to a higher dialysis efficacy of the daily schedule.

An alternative explanation for our findings would be that there was spontaneous recovery from the low-turnover bone disease and spontaneous elimination of aluminum from the bone tissue. The long-term evolution of the low-turnover bone disease is still disputed.^25^ However, in a previous report of a bone biopsy study made on eleven patients with adynamic bone disease at the start of their dialysis treatment and then after 16.6 months (SD 2.2) on maintenance dialysis, only one patient developed mild hyperparathyroidism.^26^

## CONCLUSIONS

Patients treated by short daily dialysis had slightly better control of serum phosphorus and lower Ca x P product. More importantly, the treatment was associated to an improvement of low-turnover bone disease and aluminum deposition allowing us to ponder whether these disorders could be ameliorated by more efficient dialysis alternatives.
